# Protocol for detecting oral squamous cell carcinoma in histopathology images using the momentum contrast framework

**DOI:** 10.1016/j.xpro.2025.103937

**Published:** 2025-07-31

**Authors:** Xiaoyun Zhang, Yue Fang, Weibin Liao, Junyi Ma, Xin Gao, Min Gao, Junfeng Zhao

**Affiliations:** 1 School and Hospital of Stomatology, Peking University, Beijing 100081, China; 2 School of Computer Science, Peking University, Beijing 100871, China; 3 Key Laboratory of High Confidence Software Technologies, Ministry of Education, Peking University, Beijing 100871, China

**Keywords:** Cancer, Computer sciences

## Abstract

The detection of oral squamous cell carcinoma (OSCC) in histopathology images is crucial for improving diagnostic accuracy and patient outcomes. Here, we present a protocol for detecting OSCC in histopathology images using transfer learning. We describe steps for installing software and prerequisites, preparing datasets, and pretraining a model on images from various tissue types using the momentum contrast (MoCo) framework. We then detail procedures for evaluating the fine-tuned HistoMOCO model’s performance on a test dataset.

## Highlights

• Pretrain the model with diverse histopathology datasets using MoCo for OSCC detection
• Fine-tune the model with OSCC-specific datasets to improve image classification
• Use rotation, scaling, and augmentation techniques to enhance model robustness

## Abstract (editor highlights)

Publisher’s note: Undertaking any experimental protocol requires adherence to local institutional guidelines for laboratory safety and ethics.

## Graphical abstract

**Figure:**
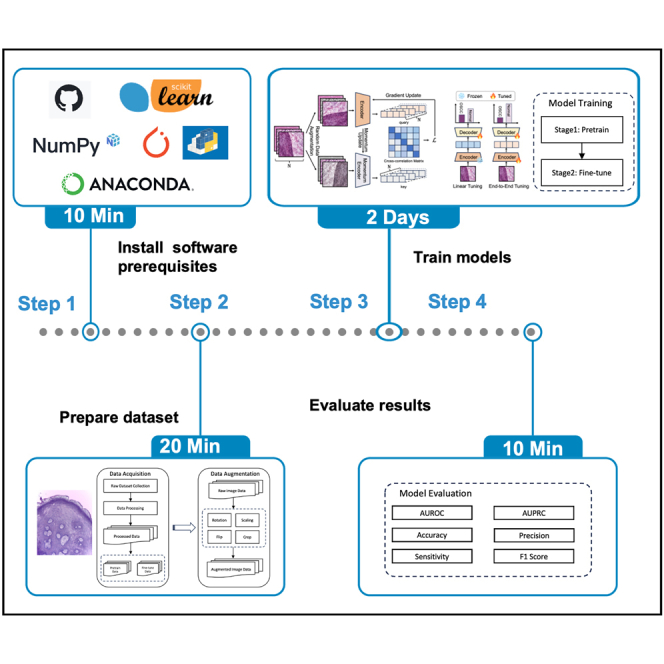

## Before you begin

Oral squamous cell carcinoma (OSCC) is the most common type of oral cancer worldwide, with high mortality and morbidity rates, particularly in regions such as Asia and Europe.^1^ Although OSCC is prevalent across numerous regions, including parts of Asia, Europe, and beyond, it continues to be insufficiently studied. This lack of comprehensive research has contributed to the persistently high rates of both mortality and morbidity associated with the disease.^2^ This highlights the critical need for early detection, as survival rates for OSCC can reach 80%–90% when diagnosed early.^3^ In contrast, late-stage diagnoses frequently result in poor five-year survival rates, often falling below 20%.^4^^,^^5^ Histopathological analysis remains the gold standard for diagnosis, but manual methods are time-consuming and prone to inconsistencies among pathologists.^6^ These limitations have spurred interest in computer-aided diagnosis (CAD) systems, particularly those utilizing deep learning, which could automate and improve diagnostic accuracy by reducing human error and ensuring consistent results.

Recent advances in machine learning, especially deep learning techniques, have shown promise for OSCC detection in histopathological images. Methods such as gray-level co-occurrence matrices (GLCM), support vector machines (SVM), and convolutional neural networks (CNNs) have been explored.^7^^,^^8^ Additionally, hybrid methods combining CNNs and traditional feature extraction techniques also show potential for improving accuracy.^9^ However, these models often suffer from limitations such as overfitting to specific tissue types or insufficient labeled data for training.

**Figure 1:**
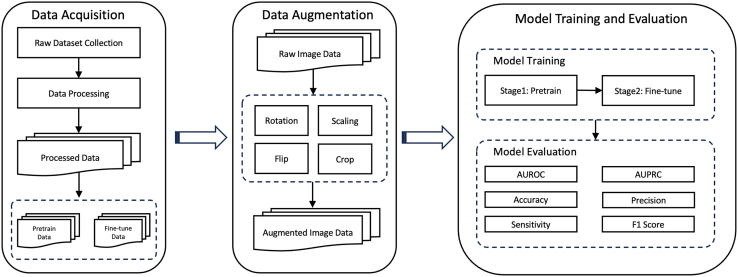
The schematic diagram of this protocol

To address these limitations, we propose a novel approach that uses the **MoCo** framework to pretrain deep learning models on histopathological images from diverse tissue types. This cross-tissue pretraining strategy aims to learn more robust and transferable features that can be fine-tuned for **detecting OSCC** versus non-cancerous tissue from histopathology images. By leveraging data from various tissue types during pretraining, the model is expected to learn generalizable features that can be effectively fine-tuned for OSCC detection, even with limited labeled data. This approach is expected to improve model performance and provide a more generalizable solution for OSCC detection across different clinical settings. The schematic diagram of this protocol is shown in Figure 1.

### Software prerequisites and requirements

This protocol requires a Linux system. Before running the experiments, the following software must be installed: Python (>= v.3.9), PyTorch (>= v.1.4.0), torchvision (>= v.0.5.0), CUDA (for GPU acceleration) with the appropriate version for your hardware, PIL (>= v.7.0.0) for image processing.

***Note:*** For optimal performance, this framework should be run on a multi-GPU setup, preferably with 4 or more high-performance GPUs, such as NVIDIA V100 or RTX series. We recommend using at least an 8-GPU RTX3090 machine for pretraining.

## Key resources table

**Table:** 

REAGENT or RESOURCE	SOURCE	IDENTIFIER
**Deposited data**		

Histopathology images for OSCC	This paper	N/A

**Software and algorithms**		

Python (>=3.9)	Python Software Foundation	https://www.python.org/downloads/release/python-365/
PyTorch (>=1.4.0)	Facebook AI Research	https://pytorch.org/
torchvision (>=0.5.0)	PyTorch Team	https://pytorch.org/docs/stable/torchvision/index.html
CUDA Toolkit	NVIDIA Corporation	https://developer.nvidia.com/cuda-toolkit
MoCo (Momentum Contrast)	Facebook AI Research	https://github.com/facebookresearch/moco
PIL (>=7.0.0)	Python Imaging Library	https://pypi.org/project/Pillow/
Matplotlib (>=2.0)	Matplotlib community	https://matplotlib.org/
PyYAML (>=3.12)	Community project	https://pypi.org/project/PyYAML/

**Other**		

NVIDIA V100 or RTX3090 GPUs	NVIDIA Corporation	https://www.nvidia.com/

## Materials and equipment

The implementation of this protocol was developed and tested on an **Ubuntu Linux** system using the **Python** programming language (>=v.3.9). All experiments, including model pretraining and fine-tuning, were carried out on a high-performance multi-GPU setup. Detailed specifications of the computational resources used are outlined in Table 1 below.

**CRITICAL:** The MoCo framework utilized in this protocol can be computationally demanding, particularly when training on large histopathology datasets. While the resources mentioned in Table 1 allow for efficient training and evaluation, users may need to adjust hardware configurations based on the size of their own data and specific experimental requirements.

***Alternatives:*** The model can run on systems with fewer GPUs or less memory, though this will significantly increase training times, especially for large datasets. Users may adjust hardware configurations according to their experimental needs.

**Table 1:** Computation resources used in this study

Operating system	Version
Ubuntu	20.04
CPU information	
RAM Memory	1.5 TB
Total number of cores	Intel(R) Xeon(R) Platinum 8468V 48 core
Processor speed	3800.0000 MHZ
GPU information	
NVIDIA RTX3090 GPU	X8
GPU memory	24G X 8
Disk storage	
SSD	2 TB

## Step-by-step method details

### Install the prerequisites

**Timing: <10 min**

**Figure 2:**
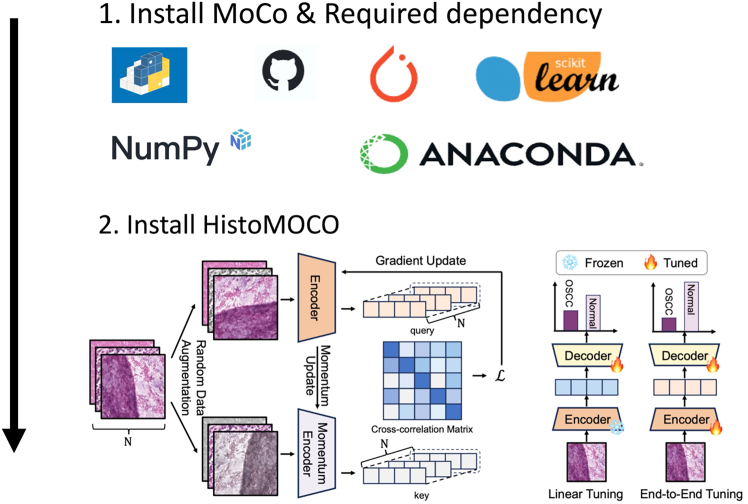
Model installation from GitHub and required dependency

This section covers the installation of required software dependencies to set up the environment for running the protocol, as shown in Figure 2.

1. Install the latest version of MOCO via pip with the following command:

>git clone https://github.com/facebookresearch/moco.git

>pip install -r requirements.txt

**CRITICAL:** It is critical to check that all required dependencies listed in the key resources table are properly installed.

***Alternatives:*** You can manually install the environment using the following methods:

2. **Install Python (>= 3.9)**: A high-level programming language essential for running the code.

***Note:*** It is highly recommended to create a virtual conda environment to avoid version conflicts. You can do this with the following command:

>conda create -n YourEnvName python=3.9

>conda activate YourEnvName

3. **Install PyTorch (>= 1.6)**: An open-source machine learning library that provides tools for tensor computation and deep learning using the following code:

>pip install torch>=1.6 torchvision torchaudio --index-url

>https://download.pytorch.org/whl/cu117

***Note:*** Ensure you have the version compatible with CUDA if using GPU acceleration. For manual download, check: https://pytorch.org/.

4. **Install NumPy (>= 1.18)**, A fundamental package for array processing in Python, crucial for numerical operations, with the following command:

>pip install numpy>=1.18

5. **Install Scikit-learn (>= 0.24)**, A machine learning library providing simple and efficient tools for data mining and data analysis, with the following command:

>pip install scikit-learn>=0.24

6. Install the latest version of HistoMOCO and via git clone:

>git clone https://github.com/Heyffff/HistoMoCo

>pip install -e.

***Note:*** Ensure that all required dependencies listed in the key resources table are properly installed to avoid errors in the installation of HistoMOCO (see troubleshooting 1). MoCo prerequisites, including Python, PyTorch, NumPy, and Scikit-learn, are essential for a successful installation process. Missing or incompatible dependencies may interrupt the installation; manual installation of any missing packages using pip or conda can resolve these issues (see troubleshooting 1).

***Note:*** When cloning the MoCo or HistoMOCO repository using git clone, ensure stable network connectivity and proper permissions to access the GitHub repository. Should cloning fail, manually download the repository as a zip file from the repository webpage and proceed with installation as instructed (see troubleshooting 2). PyTorch installation may fail, particularly when selecting a version compatible with CUDA. In these cases, use the official PyTorch website (https://pytorch.org/) to download the appropriate version.

***Note:*** If the installation fails due to network or compatibility issues, downloading the installer locally and uploading it to the computational environment for offline installation can resolve the issue (see troubleshooting 3).

### Prepare data

**Timing: <20 min**

This section covers the preparation of datasets for model training, including downloading and preprocessing images for augmentation.

In this step, we will utilize the following datasets for our model training:

First, we provide an overview of the histopathological datasets used in HistoMOCO in the Table 2 below, summarizing key characteristics. Detailed information on each dataset will follow.

7. Download EBHI-SEG: https://figshare.com/articles/dataset/EBHISEG/21540159/1?file=38179080 dataset via wget:

>wget https://figshare.com/articles/dataset/EBHISEG/21540159/1?file=38179080

**Figure 3:**
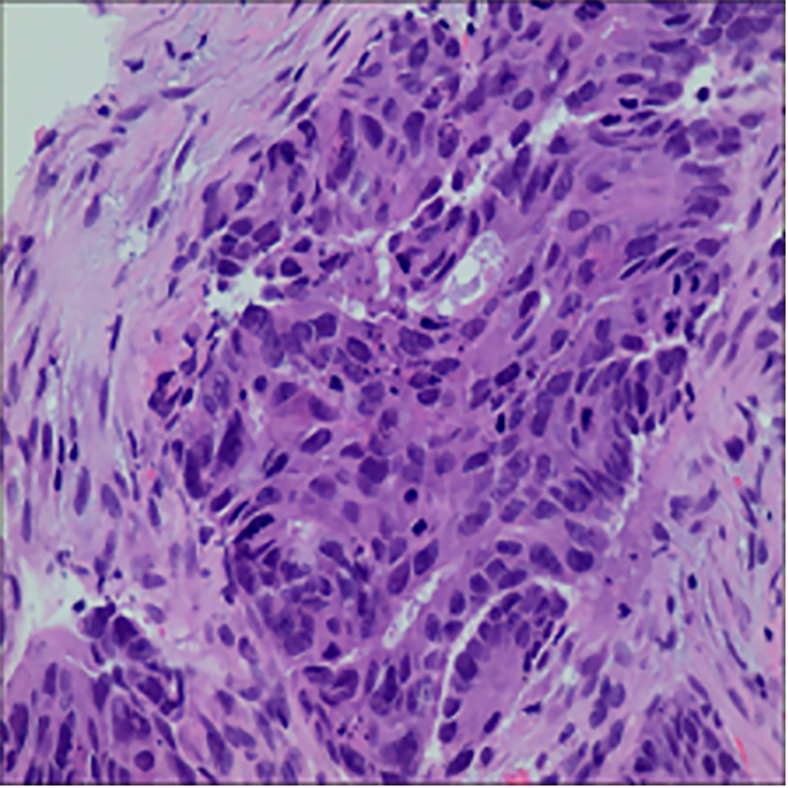
Demonstration of dataset EBHI-SE The microscopy images shown were obtained from external databases that do not provide explicit information regarding scale bars or pixel resolution. Therefore, precise scale measurements cannot be indicated for these images.

***Note:*** This dataset^10^ is designed for image segmentation tasks and contains 4,456 H&E-stained images, as shown in Table 3. It includes six categories of histopathological tissue slices along with their corresponding ground truth annotations, as shown in Table 4. An example of this dataset is shown in Figure 3.

***Alternatives:*** You can download this dataset manually via the url**:**https://figshare.com/articles/dataset/EBHI-SEG/21540159/1?file=38179080.

8. Download NCT-CRC-HE-100K: https://zenodo.org/records/1214456 dataset via wget:

**Table 3:** Statistical information of EBHI-SE dataset

Dimension	Version modality	Staining method	Anatomical structure	Anatomical region	Number of classes	Data volume	File format
2D	pathology	H&E	Colorectal	Colorectal	6	44456	.png

**Table 4:** Label distribution information of EBHI-SE dataset

Classes	Normal	Polyp	Low-grade in	High-grade in	Serrated adenoma	Adenocarcinoma
Occurrences	152	948	1278	372	116	1590
Percentage of Total	3.41%	21.27%	28.68%	8.35%	2.60%	35.68%

>wget https://zenodo.org/records/1214456

**Figure 4:**
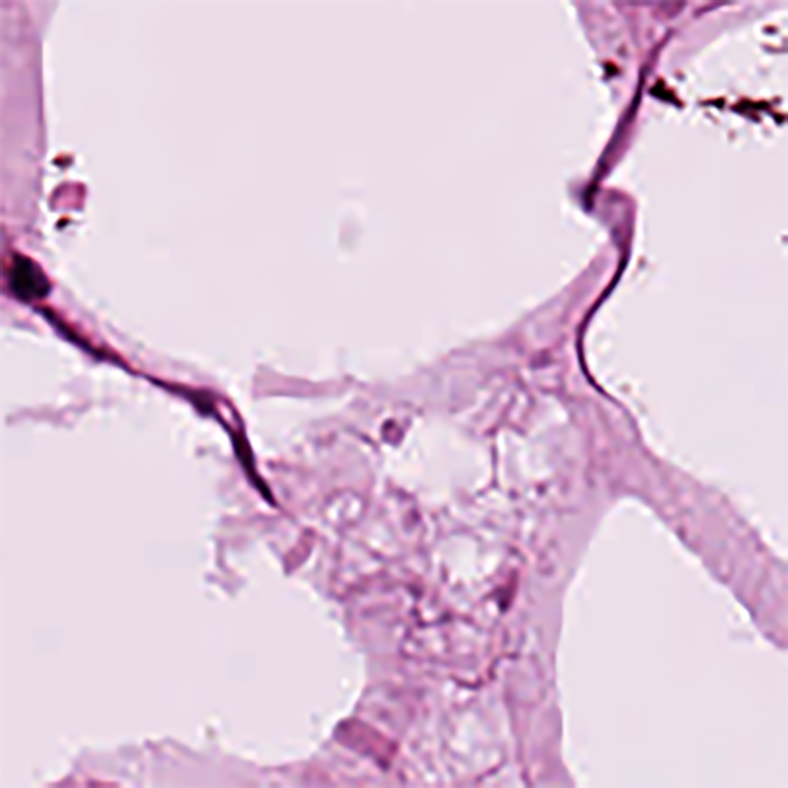
Demonstration of dataset NCT-CRC-HE-100K The microscopy images shown were obtained from external databases that do not provide explicit information regarding scale bars or pixel resolution. Therefore, precise scale measurements cannot be indicated for these images.

***Note:*** NCT-CRC-HE-100K^11^ is a specialized pathology dataset for image classification, comprising 100,000 H&E-stained histological images extracted from 86 patients with colorectal cancer and healthy tissues, as shown in Table 5. The dataset features nine distinct types of tissue images, all subjected to Macenko color normalization to minimize color variations from different slides, as shown in Table 6. This dataset will also contribute to the pretraining. An example of this dataset is shown in Figure 4.

***Alternatives:*** You can download this dataset manually via the url: https://zenodo.org/records/1214456.

9. Download NDB-UFE: https://data.mendeley.com/datasets/ftmp4cvtmb/1 dataset via wget:

**Table 5:** Statistical information of NCT-CRC-HE-100K dataset

Dimension	Version modality	Staining method	Anatomical structure	Anatomical region	Number of classes	Data volume	File format
2D	pathology	H&E	Colorectal	Colorectal	5	25000	.jpeg

**Table 6:** Label distribution information of NCT-CRC-HE-100K dataset

Classes	ADI	Back	DEB	LYM	MUC	MUS	NORM	STR	TUM
Occurrences	10407	10566	11512	11557	8896	13536	8763	10446	14317
Percentage of Total	10.40%	10.56%	11.51%	11.55%	8.89%	13.53%	8.76%	10.44%	14.31%

>wget https://data.mendeley.com/datasets/ftmp4cvtmb/1

**Figure 5:**
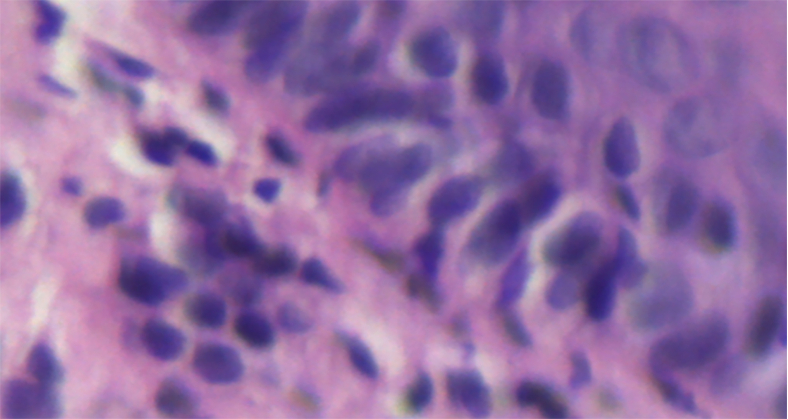
Demonstration of dataset NDB-UFE The microscopy images shown were obtained from external databases that do not provide explicit information regarding scale bars or pixel resolution. Therefore, precise scale measurements cannot be indicated for these images.

***Note:*** This dataset^12^ consists of 1,224 images divided into two groups with varying resolutions. The first group includes 89 images of normal oral epithelium and 439 images of OSCC at 100× magnification. The second group contains 201 normal epithelium images and 495 OSCC images at 400× magnification. These images were captured using a Leica ICC50 HD microscope from H&E-stained tissue slides collected and prepared by medical experts from 230 patients. Both resolution groups will be employed in downstream classification tasks. An example of this dataset is shown in Figure 5.

***Alternatives:*** You can download this dataset via the url: https://data.mendeley.com/datasets/ftmp4cvtmb/1.

10. Perform image preprocessing and augmentation.

>histomoco preprocess

>--input_dir /path/to/your/data

>--output_dir /path/to/processed/data

>--rotate 15

 --flip horizontal vertical ∖

 --scale 0.8 1.2 ∖

 --crop_size 224 224

***Note:*** The main parameters and usage are shown as follows:

 --input_dir [INPUT_DATA_PATH]: Specify the path to the directory containing the raw dataset.

 --output_dir [OUTPUT_DATA_PATH]: Specify the path where the processed data will be saved.

 --rotate [DEGREES]: Sets the degree range for random rotations; for example, --rotate 15 will rotate images randomly within ±15 degrees.

 --flip [horizontal|vertical]: Applies horizontal and/or vertical flipping; specify one or both for added variety.

 --scale [MIN MAX]: Sets the scaling range; for example, --scale 0.8 1.2 scales images between 80% and 120% of their original size.

 --crop_size [WIDTH HEIGHT]: Defines the output size after cropping; for instance, --crop_size 224 224 will crop images to 224×224 pixels.

**CRITICAL:** Verify that all dataset download links are functional before starting. If links to the **EBHI-SEG** or **NCT-CRC-HE-100K** datasets are broken or inaccessible, first confirm that the URLs are correct and check for any website maintenance notifications. Alternatively, contact the corresponding dataset authors or repository maintainers for access (see troubleshooting 4).

***Note:*** During data preprocessing and augmentation, ensure that all images are in the correct format, as specified for each dataset. **EBHI-SEG** images should be in .png format, and **NCT-CRC-HE-100K** images should be in .jpeg format. If preprocessing fails due to unsupported file formats or corrupted files, consider re-downloading the datasets or converting files to the correct format using tools such as ImageMagick or OpenCV (see troubleshooting 5).

**Table 2:** Statistical information of histopathological datasets used in HistoMOCO

Dataset	Resolution	Task	Images	Targets
**Used forpre-training of HistoMOCO**				

EBHI-SEG	224 × 224	segmentation	4,456	6
NCT-CRC-HE-100K	224 × 224	classification	100,000	9

**Used forfine-tuning of HistoMOCO**				

NDB-UFE	512 × 512	classification	1,224	2

### Train HistoMOCO model

**Timing: <2 days**

This section covers the process of training the HistoMOCO model, including initializing the model structure, pretraining with histopathology datasets using contrastive learning, and fine-tuning the model for oral cancer classification.

11. Initialize model structure:

***Note:*** You can use this command to initialize the model structure with a specified architecture and preprocessed dataset, saving the model at a defined output path. This setup also allows you to freeze the backbone layers, preparing the model for fine-tuning in subsequent training steps.

>histomoco init_model

>--data_path /path/to/preprocessed/data

>--output_path /path/to/save/model

>--model_name ResNet50

 --freeze_backbone True

 --flip horizontal vertical ∖

 --scale 0.8 1.2 ∖

 --crop_size 224 224

***Note:*** The main parameters and usage are shown as follows:

 --data_path [DATA_PATH]: Specify the path to the preprocessed dataset, which will be used in model initialization.

 --output_path [OUTPUT_PATH]: Path where the initialized model structure will be saved.

 --model_name [MODEL_NAME]: Name of the model architecture to initialize, such as ResNet50.

 --freeze_backbone [True|False]: If set to True, freezes the backbone layers of the model, keeping them untrainable, and prepares the model for training only the classifier layers.

***Note:*** The core of our study is an enhanced model based on the Momentum Contrast (MoCo) framework. MoCo is a contrastive learning method designed to build a dynamic dictionary through momentum updates, allowing the model to learn meaningful feature representations in an unsupervised manner. To tailor MoCo for the task of oral cancer classification, we made the following improvements: (1) **Encoder Replacement**: We replaced the original encoder in MoCo with ResNet18. ResNet18 is a shallower residual network with fewer parameters and faster computational speed, making it well-suited for processing medical images. Compared to deeper networks, ResNet18 demonstrated better generalization capabilities in our experiments, especially when dealing with limited data. (2) **Classifier Head**: After feature extraction, we directly employed ResNet18 as the classifier head to classify the extracted features. The fully connected layer of ResNet18 outputs are used to calculate the loss and perform backpropagation, updating the entire model’s parameters.

12. Pretrain HistoMOCO model, use the following command for pre-training:

>histomoco pretrain

>--data /path/to/pretrain/dataset

>--model ResNet50

>--init_model_path /path/to/initialized/model

>--epochs 200

>--learning_rate 30

>--batch_size 256

>--split 8:1:1

>--validation_metric auroc

>--save_best_checkpoint

>--epochs 200

>--learning_rate 30

>--batch_size 256

>--split 8:1:1

>--validation_metric auroc

>--save_best_checkpoint

***Note:*** In the pre-training phase, contrastive learning is performed on unlabeled histopathology images using the HistoMoCo framework to capture meaningful feature representations. The **EBHI-SEG** and **NCT-CRC-HE-100K** datasets are used, creating positive and negative sample pairs to develop an effective feature space.

**CRITICAL:** The main parameters and usage are shown as follows:

 --data [DATA_PATH]: Path to the pre-training dataset.

 --model [MODEL_NAME]: Model architecture for pre-training (e.g., ResNet50).

 --init_model_path [PATH]: Path to the initialized model to be loaded for pre-training.

 --epochs 200: Number of training epochs for pre-training.

 --learning_rate 30: Learning rate for the linear classifier.

 --batch_size 256: Batch size for training.

 --split 8:1:1: Specifies the split ratio for training, validation, and test sets.

 --validation_metric auroc: Uses AUROC as the metric for validation. ix.

 --save_best_checkpoint: Saves the model checkpoint with the best validation performance.

**CRITICAL:** When initializing the model structure and pre-training HistoMOCO, ensure the computational environment has adequate memory. Large batch sizes may cause the training process to crash due to memory limitations (see troubleshooting 6). Adjusting the batch size to a smaller value (e.g., 128 or 64) can alleviate this issue. If further reduction is needed, consider using gradient accumulation techniques to simulate larger batch sizes while staying within memory limits. During fine-tuning, carefully monitor memory usage, especially when using a pre-trained model and handling multiple datasets. Fine-tuning on high-resolution images or large datasets may also require memory optimization to avoid crashes.

***Note:*** The use of the EBHI-SEG and NCT-CRC-HE-100K datasets for pre-training was primarily driven by the availability of large-scale, high-quality histopathological images that allow for the learning of generalizable feature representations. Although the cancer types differ (e.g., colorectal adenocarcinoma vs OSCC), both datasets share essential histological features that can help capture robust representations applicable across various cancer types. These representations are then fine-tuned on OSCC-specific datasets, improving the model’s performance in OSCC detection despite the differences in cancer types.

13. Fine-tune HistoMOCO Model, use the following command for fine-tuning:

>histomoco finetune

>--data /path/to/fine_tune/dataset

>--model ResNet50

>--init_model_path /path/to/pretrained/model

>--learning_rate 0.001

>--save_best_checkpoint

***Note:*** In the fine-tuning phase, the pre-trained HistoMOCO model is further optimized on labeled oral cancer images from the **NDB-UFES** dataset. The dataset is split into 80% for training and 20% for testing. Using parameters from the pre-trained model as initial values, fine-tuning focuses on the classifier layer to refine classification performance without modifying the backbone, thus preventing overfitting.

**CRITICAL:** The main parameters and usage are shown as follows:

 --data [DATA_PATH]: Path to the fine-tuning dataset.

 --model [MODEL_NAME]: Model architecture used for fine-tuning.

 --init_model_path [PATH]: Path to the pre-trained model to be loaded for fine-tuning.

 --learning_rate 0.001: Sets a smaller learning rate for fine-tuning to control overfitting.

### Evaluate HistoMOCO model

**Timing: 10 min**

This section focuses on evaluating the fine-tuned HistoMOCO model’s performance on a test dataset, using different metrics to assess classification effectiveness.

14. Evaluate the Fine-tuned Model using the following command:

>histomoco evaluate

>--data /path/to/test/dataset

>--model ResNet50

>--checkpoint /path/to/best_checkpoint

>--metrics auroc,auprc

***Note:*** After fine-tuning, evaluate the model's performance on the test set, which is split from the original dataset (80% for training and 20% for testing),to assess classification effectiveness. This evaluation is performed using two approaches—linear classifier evaluation and end-to-end fine-tuning.

**CRITICAL:** The main parameters and usage are shown as follows:

 --data [TEST_DATA_PATH]: Path to the test dataset for final evaluation.

 --model [MODEL_NAME]: Specifies the model name used during training.

 --checkpoint [BEST_CHECKPOINT_PATH]: Path to the best model checkpoint, selected based on validation AUROC during training.

 --metrics auroc,auprc: Uses AUROC (Area Under the Receiver Operating Characteristic curve) and AUPRC (Area Under the Precision-Recall Curve) to evaluate classification performance.

## Expected outcomes

**Figure 6:**
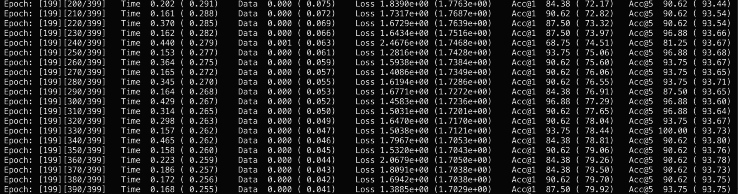
The expected outcomes after pretraining phase on the Linux terminal

**Figure 7:**
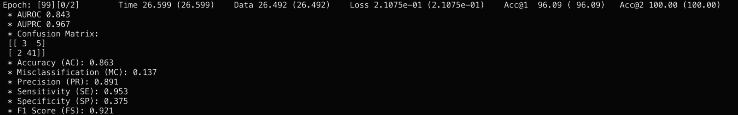
The expected outcomes after fine-tuning phase on the Linux terminal

Upon executing the proposed training procedures, we anticipate the following outcomes, as shown in Figures 6 and 7: (1) **Saved Models**: Five distinct models, resulting from the five-fold cross-validation process, will be stored in the directory ./models/. These models can be easily reloaded using the standard pickle module in Python, allowing for further analysis or deployment. (2) **Evaluation Results**: Comprehensive evaluation metrics, including accuracy, AUROC, and AUPRC from the cross-validation, will be documented in the file ./results/eva.tsv. This file will facilitate a clear assessment of the model performance across different folds. These outcomes will collectively enhance our understanding of the model’s decision-making process, guiding future iterations and applications of the developed framework in oral cancer classification tasks.

## Quantification and statistical analysis

This protocol evaluates the performance of the OSCC detection model using several widely-adopted metrics for binary classification tasks. The aim is to assess the model’s general effectiveness and its ability to distinguish between positive (OSCC) and negative (non-OSCC) cases, ensuring that the model performs robustly in real-world clinical scenarios. We have selected a combination of metrics that provide a comprehensive evaluation of the model across different aspects of classification performance. These include:

1. AUROC (Area Under the Receiver Operating Characteristic Curve):The ROC curve plots the true positive rate (TPR, or sensitivity) against the false positive rate (FPR). The AUROC score quantifies the overall ability of the model to discriminate between classes.

$$\text{TPR}=\frac{\text{TP}}{\text{TP}+\text{FN}}$$

$$\text{FPR}=\frac{\text{FP}}{\text{FP}+\text{TN}}$$

TP: True Positive, TN: True Negative, FP: False Positive, FN: False Negative.

An AUROC value of 0.5 indicates no discriminative power (random chance), while 1 represents perfect classification.

2. AUPRC (Area Under the Precision-Recall Curve):The precision-recall curve plots precision versus recall, particularly useful for imbalanced datasets. AUPRC is effective when the positive class is much smaller than the negative class, which is common in medical datasets.

$$\text{Precision}=\frac{\text{TP}}{\text{TP}+\text{FP}}$$

$$\text{Recall}=\frac{\text{TP}}{\text{TP}+\text{FN}}$$

Higher AUPRC values indicate better performance in distinguishing positive cases, particularly in imbalanced data.

3. Accuracy: Accuracy measures the proportion of correctly predicted instances (both true positives and true negatives) over the total number of instances.

$$Accuracy=\frac{\text{TP}+\text{TN}}{\text{TP}+\text{TN}+\text{FP}+\text{FN}}$$

While accuracy is a simple metric, it can be misleading in imbalanced datasets, where AUROC and AUPRC are preferred.

4. F1 Score: The F1 score is the harmonic mean of precision and recall, providing a single metric that balances both measures. It is particularly useful when the goal is to balance precision and recall.

$$F1=2\times \frac{\text{Precision}\times \text{Recall}}{\text{Precision}+\text{Recall}}$$

An F1 score of 1 indicates perfect precision and recall, while 0 indicates complete failure.

5. Specificity: Specificity measures the model’s ability to correctly identify negative cases. It complements sensitivity in evaluating model performance.

$$\text{Specificity}=\frac{\text{TN}}{\text{TN}+\text{FP}}$$

Critical Consideration: Given the nature of histopathology data and the imbalance in positive and negative cases (e.g., cancerous vs. non-cancerous), AUPRC and AUROC are particularly valuable for evaluating the model’s performance. Accuracy alone may not provide a complete picture, especially when the negative class is dominant.

## Limitations

A key limitation of our protocol is its high computational resource demand. Data preprocessing and feature extraction can significantly increase memory usage, especially with large datasets. Additionally, the reliance on deep learning models requires substantial GPU resources, which may extend training times in environments with limited capacity. Variations in image quality and dataset diversity can also affect model performance, highlighting the importance of thorough validation on new data sources.

Another limitation is the lack of exploration into model explainability, which is crucial for clinical applications. Although our approach leverages cross-tissue pretraining, the interpretability of the model’s decision-making process, particularly when working with different carcinoma types, remains a challenge. While this aspect was not the focus of this protocol, it will be an important area for future work, where techniques like grad-CAM^13^ and Saliency Maps^14^ could be incorporated to enhance model explainability.

## Troubleshooting

### Problem 1

Related to step 1, installation of MoCo due to uninstalled prerequisites.

### Potential solution

Ensure all required dependencies for MoCo are installed. First, refer to the key resources table for a list of the required libraries or software. Install these manually using package managers such as pip or conda. For example, use pip install <library_name> or conda install <library_name>. Once all dependencies are installed, retry the MoCo installation. If issues persist, verify the system’s compatibility with the specified versions of the dependencies.

### Problem 2

**Figure 8:**
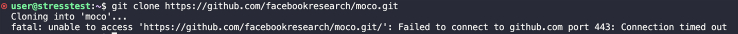
Screenshot of the terminal if the git clone command fails

The git clone command fails when attempting to clone the repository (step 1), as shown in Figure 8.

### Potential solution

If the git clone command continues to fail, manually download the repository as a zip file directly from the repository’s webpage. Navigate to the repository URL in your web browser, click on the “Code” button, and select “Download ZIP.” After downloading, extract the contents to your desired directory and proceed with the installation by following the instructions in the README file or relevant documentation. Once all dependencies are installed, retry the MOCO installation. If issues persist, verify the system’s compatibility with the specified versions of the dependencies.

### Problem 3

**Figure 9:**
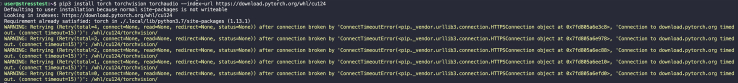
Screenshot of the terminal if the installation of Pytorch fails

The installation of PyTorch fails, especially when trying to install the version compatible with CUDA (step 1), as shown in Figure 9.

### Potential solution

Manually download the appropriate version of PyTorch from the official website at https://pytorch.org/. Select the correct version for your system, including CUDA support if using a GPU. If the failure is due to network connectivity issues, download the installation package on a local machine, then manually upload the package to the server. Once uploaded, install PyTorch using the offline installer through commands specific to your environment (e.g., conda or pip).

### Problem 4

Data download links for the EBHI-SEG or NCT-CRC-HE-100K datasets are broken or inaccessible (step 2).

### Potential solution

If the dataset download links fail, first verify the URL and check for any website maintenance or restrictions. Alternatively, contact the corresponding author for access to raw data. For EBHI-SEG, you can try requesting the dataset via Figshare, and for NCT-CRC-HE-100K, access may be possible via Zenodo or by reaching out to the dataset maintainers directly.

### Problem 5

Preprocessing fails during data augmentation due to unsupported file formats or corrupted images (step 2).

### Potential solution

Ensure that all images are in the correct format (e.g., .png for EBHI-SEG and .jpeg for NCT-CRC-HE-100K). If corrupted files are detected, re-download the datasets or manually convert the images to the appropriate format using tools like ImageMagick or OpenCV. Additionally, run a preliminary check on file integrity before proceeding with augmentation. Please note that in the ‘.py’ files located in the “dataset” folder of the HistoMoCo repository, there are references to specific dataset paths such as “original/NCT-CRC-HE-100K”, “original/EBHI-SEG”, etc. Users should ensure that the dataset paths are correctly specified, as mismatches in naming could lead to errors during execution.

### Problem 6

**Figure 10:**
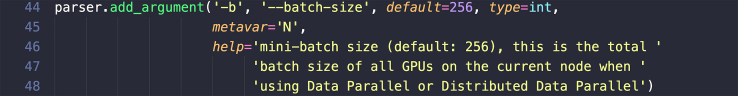
Default parameter setting for batch size

The model training process crashes due to insufficient memory, particularly when handling large batch sizes during training (step 3). The screenshot shown in Figure 10 corresponds to the adjustment of the ‘batch-size’ parameter in the ‘pretrain.py’ file to mitigate memory issues during training.

### Potential solution

Reduce the batch size parameter (e.g., --batch_size 256) to a smaller value like 128 or 64 to accommodate the available memory. If this is insufficient, consider using gradient accumulation techniques to mimic larger batch sizes without overloading the memory, or run the training on a machine with more GPU memory.

## Resource availability

### Lead contact

For additional details or inquiries, please reach out to the lead contact, Xiaoyun Zhang (xiaoyunzhangpku@126.com).

### Technical contact

Technical questions on executing this protocol should be directed to and will be answered by the technical contact, Xiaoyun Zhang (xiaoyunzhangpku@126.com).

### Materials availability

This study did not generate new unique reagents.

### Data and code availability

This study uses open-source datasets, with their sources appropriately cited in the manuscript. The code used in this study is available at [https://github.com/Heyffff/HistoMoCo]. https://doi.org/10.5281/zenodo.14614211.

## Acknowledgments

This work was supported by Capital’s Funds for the Health Improvement and Research (no. 2024-2G-4106).

## Author contributions

Methodology, X.Z. and Y.F.; investigation, W.L. and Y.F.; funding acquisition, X.Z. and J.Z.; writing – original draft, Y.F.; writing – review and editing, J.M. and X.G.

## Declaration of interests

The authors declare no competing interests.
